# Transmission of Grapevine Leafroll-Associated Viruses and Grapevine Virus A by Vineyard-Sampled Soft Scales (*Parthenolecanium corni*, Hemiptera: Coccidae) †

**DOI:** 10.3390/v14122679

**Published:** 2022-11-29

**Authors:** Gérard Hommay, Monique Beuve, Etienne Herrbach

**Affiliations:** Unité Mixte de Recherche 1131 Santé de la Vigne et Qualité du Vin (SVQV), Université de Strasbourg, Institut National de Recherche pour l’Agriculture, l’Alimentation et l’Environnement (INRAE), F-68000 Colmar, France

**Keywords:** *Vitis vinifera*, scale insect, leafroll, rugose wood, GLRaV, ampelovirus, GVA, vitivirus, vector, virus transmission

## Abstract

Grapevine-infecting ampelo- and vitiviruses are transmitted by scale insects belonging to several species, among which is the European fruit lecanium, *Parthenolecanium corni* (Bouché) (Hemiptera Coccidae). Our objective was to characterize the transmission biology of grapevine leafroll-associated viruses (GLRaV) and grapevine virus A (GVA) by this soft scale species in order to evaluate its ability to spread these viruses. In transmission experiments with nymphs sampled from different vineyards infected with GLRaV 1, 2, 3 and GVA, *P. corni* transmitted only GLRaV 1 and GVA to healthy vines. GVA was predominantly transmitted along with GLRaV 1, whereas the latter could be transmitted alone from single or co-infected vines. Vineyard-sampled second instar nymphs were more efficient than first instars at transmitting GLRaV 1, whereas both instars displayed similar transmission rates for GVA. Short virus inoculation access periods and the absence of virus in eggs of females living on infected grapevines fulfilled the criteria of non-circulative semi-persistent transmission mode.

## 1. Introduction

One of the most important viral diseases of grapevine (*Vitis vinifera* L), ‘grapevine leafroll disease’ (GLD) occurs in all major grapevine-growing areas. In New York State, USA, mean yield losses are estimated at 15–20%, but can reach up to 40% [1]. So far, six distinct viruses belonging to the family *Closteroviridae*, namely, grapevine leafroll-associated virus (GLRaV) 1, 2, 3, 4, 7 and 13 have been associated with GLD [2,3,4,5]. Members of four among them, GLRaV 1, 3, 4 and 13, are assigned to the genus *Ampelovirus*, and are naturally transmitted to grapevine by scale insects (Hemiptera Coccoidea) belonging to the families Coccidae (soft scales) and Pseudococcidae (mealybugs) [6,7]. Notably, GLRaV 3 can also be experimentally transmitted by *Planococcus ficus* (Signoret) to the herbaceous host *Nicotiana benthamiana* Domin [8]. Moreover, these scale insects are able to transmit three virus species associated with the ‘rugose wood complex’ of grapevine and members of the genus *Vitivirus*: grapevine virus A (GVA), grapevine virus B (GVB) and grapevine virus E (GVE) [7]. These ampelo- and vitiviruses are restricted to grapevine phloem tissues. GVA is transmitted to *Nicotiana benthamiana* and *N. clevelandii* Gray by scale insects of three species: *Pl. ficus* [9], *Pseudococcus longispinus* (Targioni Tozzetti) [10] and *Parthenolecanium corni* (Bouché) [11]. As these vitiviruses are frequently transmitted along with GLRaV 1 or 3, the hypothesis that ampeloviruses may assist vitiviruses during transmission has been raised [7,11,12,13,14]. A semi-persistent transmission mode is believed to apply to soft scales, as already shown for mealybugs (e.g., [7,10,15,16,17,18,19,20]). Indeed, few studies have been devoted to virus transmission by soft scales [11,12,21,22,23,24,25,26,27,28,29], due to a less mobile lifestyle and handling difficulties. This statement relies on: (1) short (i.e., from less than one to a few hours) acquisition and inoculation access periods (AAP and IAP, respectively) for an efficient virus transmission, (2) absence of a latency period, and (3) a virus retention in the vector not exceeding a few days with mealybugs either starving or feeding on a virus non-host. Moreover, as for other semi-persistently-transmitted viruses [30], the phloem-restriction of ampelo- and vitiviruses requires the vector’s stylets to reach the phloem tissue for efficient acquisition and inoculation.

Four of the aforementioned viruses are known in French vineyards: GLRaV 1, 2, 3 and GVA. In northeastern France, the palearctic soft scale *P. corni*, the European fruit lecanium, is the most widespread species in vineyards and is monovoltine. Adult females lay eggs mainly on canes in spring, and neonates, called ‘crawlers’, migrate to basal leaves. Once fixed on leaves, the nymphs move only to find a new feeding place or when unfavourable conditions of survival (drying, withering) force them to leave their attachment point, and when they molt from first (L1) to second (L2) instar nymphs during summer [31]. The main mobility periods occur during seasonal migrations: when after hatching on canes, L1 head for leaves, and when L2 migrate in the autumn towards woody parts of canes and cordons to overwinter and back to canes in early spring [31,32,33]. *P. corni* L1 can be spread by wind and thus transmit GLRaV 1 and GVA to neighboring vineyards [34,35]. However, the ability of *P. corni* natural populations dwelling on infected vines to effectively transmit the viruses is poorly known and evaluated. Therefore, our purpose was to investigate the transmission potential of vineyard-resident *P. corni* depending on its developmental stages. For this purpose, we used transmission experiments, consisting of sampling L1 and L2 nymphs, in spring and summer–autumn, respectively, from leafroll-infected grapevines in a vineyard, and transferring them onto healthy vine cuttings in the laboratory [24,25]. Such sampled nymphs are considered to harbour the virus for an AAP of undetermined duration.

## 2. Materials and Methods

### 2.1. Origin of Insects and Viruses

*P. corni* populations originated from five commercial grapevine vineyards located at Bennwihr, Kientzheim, Nothalten, Ribeauvillé and Turckheim (Alsace, northeastern France), as well as from an experimental vine vineyard located at Ihringen (*Staatliches Weinbauinstitut*, Freiburg, southwestern Germany). Distribution of viruses among the vines tested in each vineyard is given in Table 1. Heavily infested grapevines were first tested for GLRaV 1, 2, 3. and GVA by enzyme-linked immunosorbent assay (ELISA) (see Section 2.5), before sampling leaves bearing soft scales for inoculation experiments. Additional reverse transcription polymerase chain reaction (RT-PCR) tests were performed on grapevines for which ELISA results were not conclusive (see Section 2.2). Each grapevine was spatially localized (row number, grapevine number). We tried to obtain a representative number of each virus combination among virus source grapevines hosting *P. corni* colonies. GLRaV 2 being rare in our vineyards, associations with this virus were underrepresented. IAP experiments were performed from 12 June to 26 July for L1, and from 23 July to 30 October for L2, depending on annual climatic conditions.

### 2.2. Viral Content of Soft Scales and Source Sampled

A multiplex RT-PCR procedure was used for the detection of GLRaV 1, 2, 3 and GVA in insect samples at different growth stages (batches of ≥25 L1, 1 to 20 summer–autumn L2, 8 to 45 overwintering L2, and 2 to 11 maturing females) before transmission experiments, using the protocol developed by Beuve et al. [36]. In addition, ten batches of >100 freshly laid eggs taken under the shield of ten different adult females from a grapevine co-infected with GLRaV 1 and GVA were tested, as well as honeydew excreted by females fed on four different GLRaV 1 infected grapevines.

Nymphs were collected and stored in 1.5 mL Eppendorf tubes with 500 μL RLT buffer (RNeasy Plant Mini Kit™, Qiagen, Courtaboeuf, France) containing 1% β-mercaptoethanol. Tubes were kept at −20 °C before total RNA extraction and virus detection by multiplex RT-PCR. The content of the Eppendorf tubes was ground with a PolyLabo™ electric mill, then incubated for 3 min at 56 °C. A quantity of 450 μL of supernatant from the tubes was centrifuged for 2 min at 14,000 rpm in an RNeasy column tube to remove cellular debris and precipitates. Then, 400 μL of filtrate was poured in a 1.5 mL tube, to which 200 μL ethanol was added. The mix was poured in an RNeasy column tube and centrifuged for 30 s at 10,000 rpm to fix the RNA. The column was then washed with 700 μL RW1 buffer for 5 min and centrifuged for 30 s at 10,000 rpm, and then with two successive washes with 500 μL of RPE buffer and respective centrifugations of 30 s and 2 min at 10,000 rpm. Residual ethanol in tubes was removed by centrifugation for 1 min at 10,000 rpm. Total RNAs were eluted during 1 min with 50 μL RNase-free water; then, column tubes were centrifugated for 1 min at 10,000 rpm, and eluates were stored in 1.5 mL tubes at −20 °C for subsequent RT-PCR. All centrifugations were performed at room temperature. RNase-free water and RNAs extracted from leaves of the accession Y258, an Armenian cultivar Liali Bidona co-infected with GLRaV 1, 3 and GVA sampled from our collection of accessions [37], were used as negative and positive controls, respectively. 

For PCR, in each sample, 12.5 µL of “Master Mix”, 2.5 µL of “Factor Q”, 7.8 µL of sterile ultra-pure RNase free water and 1.2 µL of the mix of primers-PCR (Table 2) at 25 µM were added to 1 µL of cDNA, except for the controls. The negative controls for RT and PCR contained water instead of cDNA, and the positive control cDNA from Y258. The samples were then placed in a thermal cycler (VWR Doppio Mastercycler™, Eppendorf, Hamburg, Germany) set up for an initial denaturation step (1 cycle of 15 min at 95 °C), denaturation / hybridization / extension steps (35 cycles of 30 s at 94 °C, 1 min 30 at 56 °C; then, 1 min at 72 °C), and a final extension step (1 cycle of 30 min at 60 °C). PCR products were visualised under UV light on a 2% agarose gel stained with ethidium bromide.

In a set of source grapevines from Nothalten, the viral content of the leaves and of the L1 nymphs was analysed by RT-PCR on different canes of the same plant. A quantity of 100 mg of leaf tissue was ground at room temperature with pestle, mortar and Fontainebleau sand in 1 mL RLC buffer (RNeasy Plant Mini Kit™, Qiagen, Courtaboeuf, France), to which was added 10 μL β-mercaptoethanol and 20 mg polyvinylpyrrolidone 40. The extract was transferred to a 1.5 mL Eppendorf tube, incubated for 3 min at 56 °C and centrifuged for 1 min at 4000 rpm. RNA extraction was then performed with approximately 400 μL of supernatant, as described previously.

### 2.3. Recipient Grapevines

Grapevines free of leafroll viruses and GVA were obtained either from rooted cuttings of *V. vinifera* cv. Pinot noir, a cultivar frequently planted in Alsace, Champagne and Burgundy (clones P114 and P115), or from germinated seeds of Pinot noir, Pinot blanc and Muscat Ottonel. Mother plants of these cuttings were regularly tested for the absence of GLRaV 1, 2, 3 and GVA by ELISA and RT-PCR. Plants were grown individually in pots under greenhouse until the 6–12 leaves stage and then used in transmission experiments. They were regularly sprayed with an insecticide to ensure the absence of insects. This spraying was stopped at least one month before transmission experiments. Samples of ten recipient plants were tested for their virus-free status with ELISA prior to experiments.

### 2.4. Transmission Experiments

Leaf pieces bearing ca. 100 L1, or 50 to 100 L2, were cut from source plants and clipped onto different leaves of recipient grapevines (distributed on 3–4 leaf pieces per plant) for an inoculation access period (IAP) of 5–7 days. Within 3–4 days, as the leaf fragments dried out, most of the insects crawled off to the recipient plant. For sampling overwintering L2, twigs of infected vines bearing L2 were collected in January in the Riesling vineyard of Nothalten. The L2 were allowed to wake up at room temperature and then placed with a fine paintbrush into cages (h = 8 mm, internal diameter = 13 mm). The cages were then attached with hairclips to leaves of recipient vines grown under a heated glasshouse (*n* = 10, mean number L2/vine ± sd = 17 ± 13). Each recipient plant was isolated under a 0.1 mm–mesh perforated plastic bag (Sealed Air SAS, Épernon, France). Transmission experiments were conducted at 20–23 °C, 16 h/8 h (L/D) under artificial light. After IAP, source leaves were removed and recipient plants were immediately sprayed with mevinphos (4 mL/L Phosdrin W10™, Cyanamid Agro, Tassin-la-Demi-Lune, France) to kill the insects. After two days, the treated plants were checked for possible surviving insects, then transferred into a glasshouse compartment dedicated only to insect-free recipient plants. Recipient plants with nymphs from healthy grapevines and conducted under the same conditions were used as negative controls. In late November, the recipient plants were pruned back to two buds and stored under a glasshouse in cold conditions for hibernation. In spring, they were transferred into a heated glasshouse. All recipient plants were periodically sprayed with insecticide and fungicide, and pruned to avoid overgrowth until the end of the study.

### 2.5. Virus Detection in Recipient Plants by ELISA

Infection of recipient grapevines was checked by double-antibody sandwich (DAS)-ELISA. The accession Y258, multi-infected with GLRaV 1, 3 and GVA, was used as positive control for the three viruses. The accession Chardonnay V38 [37], infected with GLRaV 2 and 3, was used as positive control for GLRaV 2. Healthy accession P115 [38] cuttings were used as negative controls. Tissue extracts were obtained from pooled fragments of three leaves, due to the uneven distribution of virus in grapevines (one near wine stock and two on opposite canes). Leaf fragments (1 g in 5 mL buffer) were ground in extraction bags with a bullet blender (Homex 5™, Bioreba, Switzerland). Polyclonal antibodies raised against GLRaV 1, 2, 3 or GVA produced in our laboratory were used in a biotine–streptavidine procedure [39]. Absorbance was recorded at 405 nm using a Multiskan™ microplate reader (Thermo Labsystems, Helsinki, Finland). Values above the mean of healthy controls (six replicates per ELISA plate) plus 3 times their standard deviation were considered positive. Recipient grapevines were checked by ELISA ca. 4 months and 8–12 months after IAP, and up to 18–24 months for surviving plants which remained negative before. However, the plants inoculated after late September could not be tested before cold storage and were first tested ca. 6 months later. All recipient grapevines were tested systematically for GLRaV 1, 3 and GVA. GLRaV 2 was only looked for when the source grapevine was infected with this virus.

### 2.6. Study of Minimal Inoculation Access Period

The method was the same as described above (Section 2.4), with IAP spanning from 15 min to 7 h 15 min. Recipient healthy Pinot noir plants were inoculated with *P. corni* L2 nymphs sampled on grapevines from Nothalten infected with either GLRaV 1, or GLRaV 1 and GVA, or GLRaV 1, 3 and GVA. Our previous transmission experiments from these virus associations showed the best transmission rates (see Section 3.2).

### 2.7. Statistical Analysis

Chi-square tests were used to compare rates of virus transmission by *P. corni*, according to virus associations in source vines and according to larval stages. A *p*-value < 0.05 was considered as the threshold for significance. Statistical analyses were performed with R, version 2.10.

## 3. Results

### 3.1. Virus Detection in Natural Populations of P. corni

GLRaV 1, 3 and GVA were detected in all the developmental instars of *P. corni* (Table 2) sampled from infected grapevines in vineyards, but not in eggs. Only a few freshly hatched L1 batches still under female shield (3/12, Table 3), thus unlikely to have fed on phloem, were positive for viruses; alternatively, they may have been contaminated by their mother’s honeydew. GLRaV 1 was more frequently detected than GVA, where both viruses were initially present in source plants. GLRaV 2 was only detected in one L2 batch out of two. All three viruses were detected in overwintering L2. In addition, GLRaV 1 could be detected in honeydew of L2 from four different leafroll-infected grapevines.

### 3.2. Virus Transmission Experiments by Natural Populations of P. corni

Both *P. corni* L1 and L2 nymphs sampled from vineyard-infected vines transmitted GLRaV 1 and GVA (Table 4). GVA was predominantly transmitted along with GLRaV 1. One recipient vine was infected with GVA alone from a source vine infected with GLRaV 1 and GVA, and a second from a GLRaV 3- and GVA-infected source vine, but this plant died before it could be tested with RT-PCR to check for the possible presence of leafroll viruses. Transmission rates ranged from 29 (with 100 L1/recipient vine) to 42% (with 50–100 L2) for GLRaV 1, and from 27 (with 100 L1) to 36% (with 50–100 L2) for GVA. Transmission rates were significantly different between L1 and L2 for GLRaV 1 (χ^2^ = 4.15, df = 1, *p* = 0.04), with a higher transmission rate for L2. No difference in infection rate was observed for GVA (χ^2^ = 104, df = 1, *p* = 0.31). Whatever the growth stage, transmission rates between GLRaV 1 and GVA were not significantly different whether the source plants harboured GLRaV 1 and GVA, or GLRaV 1, 3 and GVA (χ^2^ test, df = 1; Table 4). Whatever the origin and the cultivar of the source grapevine (Pinot noir, Riesling or Sylvaner; Table 5) or the recipient cultivar (Pinot noir, Pinot blanc or Muscat Ottonel; Table 6), GLRaV 1 and GVA were successfully transmitted. However, transmission events to Muscat Ottonel were scarce, likely due to few replicates. Transmission events of GLRaV 1 were significantly different (χ^2^ = 16.78, df = 3, *p* = 0.001) when it was alone or associated with any other virus in the same source. Thus, GLRaV 1 transmission rate was lower when the source plant was co-infected with only GLRaV 3 (Table 5). No transmission of GLRaV 2 and 3 was observed (Table 4). Virus transmission by active nymphs was observed through almost the whole transmission experiment period (12 June to 30 October) with the first IAP on 12 June with L1, and the last on 12 October with L2. Overwintering L2 did not transmit GLRaV 1; nevertheless, out of 7–50 L2 batches placed on recipient vines, only two to six L2 settled on recipient vines, probably being disturbed by the waking up. Healthy control plants were all negative. No living insects were found after insecticide treatments of recipient plants.

For a set of transmission experiments, virus content was tested in different canes from the same source vines of Nothalten and their corresponding L1 nymphs, and compared to that of their respective recipient grapevines (Table S1). Results show that (1) if nymph batches were positive, transmission varied between canes of the same source plant, (2) whereas batches of nymphs tested negative for GVA or GLRaV 1, transmission to a recipient grapevine was observed in two cases (Table S1, source grapevines 6-2-2 and 5-72-2).

Symptoms on recipient grapevines appeared 3 to 4.5 months after inoculation, but they were not always visible on grapevines which tested positive using ELISA. The earliest transmission event of GLRaV 1 and GVA was detected 69 days (2.3 months) after inoculation. For GLRaV 1, 64% transmissions were first detected ca. 4 months, 32% ca. one year, and 4% 15 months, after inoculation. For GVA, 67% transmission were first detected ca. 4 months, 28% ca. one year, and 5% 15 months, after inoculation. Overall, detection delay of GVA appeared similar to that of GLRaV 1, although GVA could sometimes be detected earlier than GLRaV 1.

### 3.3. Time Threshold of Inoculation Access Period (IAP)

**Figure 1:**
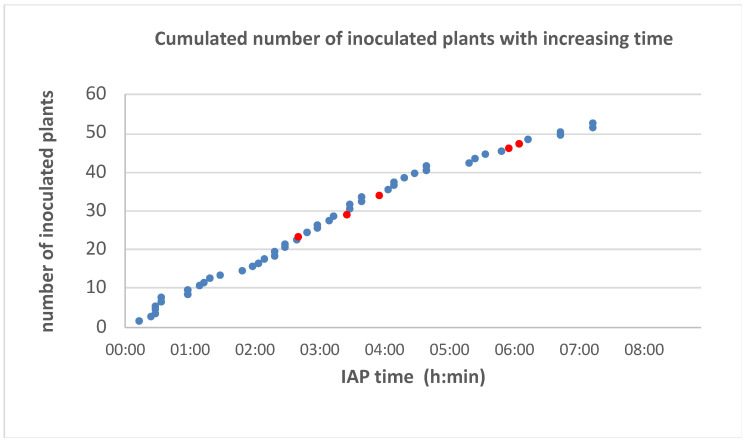
Inoculation access period durations tested for transmission of GLRaV 1 by L2 nymphs of *Parthenolecanium corni* from a natural population of Nothalten. Times for successful transmissions are indicated by red dots, and no transmissions by blue dots.

L2 nymphs collected on infected vines in Nothalten vineyard transmitted GLRaV 1 to five out of fifty-two inoculated grapevines, giving an overall transmission rate of 9.6%. The shortest IAP was 2 h 40 (Figure 1).

## 4. Discussion

Our experiments aimed at better understanding the vector biology of *P. corni* in relation to grapevine-infecting ampelo- and vitiviruses, by performing transmission experiments of nymphs sampled in infected vineyards. 

### 4.1. Virus Transmission Experiments by Natural Populations of P. corni

First (L1) and second (L2) instar nymphs were sampled on infected grapevines in vineyards. When batches of L1 and L2 were tested by RT-PCR, most of them were found harbouring viral RNA of at least one virus among GLRaV 1, 2, 3 and GVA (Table 3), confirming that they had acquired these viruses while feeding. In nymphs sampled from mixed-infected grapevines, GVA was less frequently detected than GLRaV 1 (Table 3), while there was no statistical difference between GLRaV 1 and GVA transmission rates (Table 4). Moreover, detection rates of GLRaV 1 and GVA within nymphs were higher than transmission rates to inoculated grapevines (Table 3 and Table 4). It has already been reported in mealybug species that, although nymphs showed high virus detection rates, virus transmission occurred at lower rates [15,40,41]. Even if viruses are detected in nymphs, they are not always transmitted to healthy grapevines, depending probably on factors acting on their retention at specific sites.

When L1 and L2 similarly sampled were subjected to inoculation to healthy recipient plants, they transmitted GLRaV 1 and GVA to grape cuttings, but neither GLRaV 3 nor 2 (see Section 4.2 and Section 4.3) (Table 4). Around 30% of grapevines appeared positive for viruses not earlier than one year after inoculation, underlining the need for checking for infection at least until this period. Krüger and Douglas-Smit [29] also indicated that some plants tested positive more than one year after transmission experiments.

Sforza et al. [23] previously reported that groups of 30–50 vineyard-collected *P. corni* L2 transmitted GLRaV 1 to 29% of recipient plants. With L2, we obtained a maximal transmission rate of 48% for GLRaV 1 (Table 5). Transmission rates were not significantly different between vineyard-sampled L1 and L2 of *P. corni*. Similarly, field-collected *Ph. aceris* L1 and L2 transmitted GLRaV 1 with the same efficiency [17]. Transmission rates of GLRaV 1 were significantly different when this virus was alone or associated with GLRaV 3 and GVA viruses in the source plants (Table 5). GLRaV 3 was never transmitted, but simultaneous presence of GLRaV 1 and 3 in the source plant tended to reduce GLRaV 1 transmission by *P. corni*, whereas the additional presence of GVA did not modify it; this could result from a possible competition between the two ampeloviruses for retention sites, and/or an effect of virus variability. Our results were indeed obtained from several series of transmission experiments, and these variations are probably related to the various source grapevines (locations and cultivars) and experimental conditions (developmental stages and dates). Further work should quantify virus titer in mixed infections using qRT-PCR to evaluate the virus ratio in transmission experiments.

### 4.2. Case of GLRaV 3

In our transmission experiments with *P. corni* populations sampled from several vineyards, GLRaV 3 was never transmitted (Table 4 and Table 5). Moreover, transmission experiments with insects from singly- or co-infected grapevines and conducted under the same conditions gave positive results with GLRaV 1 and negative with GLRaV 3. Globally, *P. corni* has not been shown as a vector of GLRaV 3, with the exception of a report from Washington State, USA [28], suggesting the existence of genetic variants of GLRaV 3 or/and *P. corni*. Indeed, GLRaV 3 shows a wide genetic variability and vector transmission can be affected by the virus genotype [42].

### 4.3. Case of GLRaV 2

Even though the number of samples was too small to draw a conclusion, *P. corni* was unable to transmit GLRaV 2 in our transmission experiments, though it could acquire it. Within the *Closteroviridae* family, GLRaV 2 is assigned to the genus *Closterovirus*, based on its genomic organization. While most members of this genus, including beet yellows virus (BYV), are transmitted by aphids [43,44], no vector is known so far for GLRaV 2 [7,45]. Klaassen et al. [46] suggested that the aphid *Aphis illinoisensis* Shimer might contribute to the spread of GLRaV 2 among American wild *Vitis* spp. Unintentional propagation of this virus is actually due to diffusion of infected scions and/or rootstocks.

Indeed, virus detection in individuals (viruliferousness) is not linked to their transmission ability. GLRaV 2 and GLRaV 3 were detected in nymphs, even if these viruses were not transmitted. GLRaV 1 was ingested with sap, and rejected in honeydew in which it was also detected (Table 3).

In conclusion, our transmission experiments with vineyard-sampled viruliferous nymphs showed that *P. corni* is a vector of GLRaV 1 and GVA but not of GLRaV 3, and that GVA was predominantly transmitted along with GLRaV 1 and rarely alone. Virus inoculation occurred within a few hours, in agreement with a semi-persistent transmission mode, as shown for mealybug species [7]. Further research is needed to elucidate virus–vector interactions and factors influencing the transmission efficiency and retention of viruses by scale insects, as well as potential interactions between co-transmitted viruses. In particular, further work is required to localize the receptors retaining the virions in the vector. The semi-persistent transmission mode suggests that the retention site is located in the foregut of the vector [30]. Using an immunofluorescent labeling of viruses, virions were localised at two sites in mealybug mouthparts: the cibarium [8,19] and in the tip of the stylets [8]. To confirm these observations and determine where virions are precisely retained before being released for inoculation, it will be required to locate labeled virions within histological sections of coccoids prepared for either light or electron microscopy [6]. Progress in this domain could lead to the development of innovative phytoprotection strategies by inhibiting transmission.

## Figures and Tables

**Table 1:** Location of the vineyards sampled for *Parthenolecanium corni* populations and sanitary status of vines tested in ELISA for GLRaV 1, 2, 3 and GVA. The number of source vines used for transmission tests is indicated between brackets, with one or several replicates depending on the number of nymphs available.

**Locality**	**Latitude**	**Longitude**	**Cultivar**	**Number**	**Negative**	**Positive for**							
				**of Vines Tested**		GVA	GLRaV 1	GLRaV 3	GLRaV 1, GVA	GLRaV 1, 3	GLRaV 3, GVA	GLRaV 1, 3; GVA	GLRaV 2 with 1 or 3 or GVA
Bennwihr	48°08′17.7″ N	7°19′06.6″ E	Pinot noir	142	48 (2)		4	61 (12)		21 (1)		6 (1)	2
Kientzheim	48°08′24.8″ N	7°16′20.6″ E	Riesling	24	2				22 (7)				
Nothalten	48°21′31.7″ N	7°24′39.7″ E	Riesling	704	279 (28)	1	208 (36)	24 (12)	99 (17)	45 (11)	5 (5)	33 (10)	10 (6)
Ribeauvillé	48°11′55.6″ N	7°20′11.4″ E	Riesling	21	17			2 (1)	1 (1)			1	
Turckheim	48°05′41.8″ N	7°16′34.2″ E	Sylvaner	14	2 (1)		2 (2)		9 (5)			1	
Ihringen	48°03′18.7″ N	7°37′30.0″ E	Kerner	16	4 (2)		1	11 (9)					

**Table 2:** Sequences of the primers used for the amplification of GLRaV 1, 3 and GVA by multiplex RT-PCR. The primers were designed in conserved sequences among isolates of each virus available in the GenBank (NCBI) database (Beuve et al., 2013 [36]).

Virus	Target ORF	Primer	Nucleotide Sequence 5′-3′	Hybridisation Temperature	Amplicon Length (pb)
GLRaV 1	HSP70	LR1-H70F1	GTTGGTGAATTCTCCGTTCGT	56 °C	402
		LR1-H70R1	ACTTCGCTTGAACGAGTTATAC		
GLRaV 3	Polymerase	LR3-POLF1	ACGTAACGGGGCAGAATATAGT	56 °C	282
		LR3-POLR1	TATCAACACCAAGTGTCAAGAGTA		
GVA	Coat protein	GVA-CPF1	GGCTACGACCGAAATATGTAC	56 °C	524
		GVA-CPR1	AGAAACGATGGGTCATCCATC		

**Table 3:** Viruses detected by RT-PCR in *Parthenolecanium corni* nymph batches, according to their developmental stage and to virus associations in plant sources from vineyards prior to transmission experiments. Positive detection rates are highlighted in bold. For each stage, soft scale numbers with mean number ± sd per sample (*n*).

		**Viruses in Source Plant**							
Soft scales no. and stage	Viruses detected	GLRaV 1	GLRaV 3	GLRaV 1, 3	GLRaV 1, 2, 3	GLRaV 1, 2, GVA	GLRaV 1, GVA	GLRaV 1, 3, GVA	**Total**
>100 eggs	GLRaV 1						0/10		0/10
(*n* = 10)	GVA						0/10		0/10
	GLRaV 1	**5/5**		**2/2**			**22/22**	**6/8**	**35/37**
≥25 L1	GLRaV 3			**2/2**				**3/8**	**5/10**
(47 ± 16, *n* = 37)	GVA						**14/22**	**3/8**	**17/30**
	GLRaV 1	**16/16**			0/1	**1/1**	**10/10**	**11/11**	**38/39**
1–20 L2	GLRaV 2				0/1	**1/1**			**1/2**
(8 ± 7, *n* = 43)	GLRaV 3		**1/4**		**1/1**			**4/11**	**6/16**
	GVA					**1/1**	**7/10**	**2/11**	**10/22**
8–45 L2	GLRaV 1	**8/14**		**1/1**			**7/7**	**3/3**	**19/25**
overwintering	GLRaV 3		**3/4**	**1/1**				**1/3**	**5/8**
(24 ± 9, *n* = 29)	GVA						**2/7**	0/3	**2/10**
2-11 maturing	GLRaV 1	**3/5**		**4/7**			**2/2**		**9/14**
females	GLRaV 3			**1/7**					**1/7**
(8 ± 4, *n* = 14)	GVA						0/2		0/2
25–100 L1	GLRaV 1	0/2		**2/3**			0/2	**1/5**	**3/12**
under adult	GLRaV 3			0/3				0/5	0/8
shield (*n* = 12)	GVA						0/2	**1/5**	**1/7**
L2 honeydew (*n* = 4)	GLRaV-1	**4/4**							**4/4**

**Table 4:** Virus transmission rates by vineyard-collected *Parthenolecanium corni* nymphs according to their developmental instar and to virus associations in plant source, after transmission tests: number of positive vines/number of inoculated vines. Transmission events are highlighted in bold. Overwintering L2 are marked with an *.

		**Viruses of source plants**							
No. nymphsand stage	Virus transmission	GLRaV 1	GLRaV 3	GLRaV 2 (with 1, 3 or GVA)	GLRaV 1, 3	GLRaV 1, GVA	GLRaV 3, GVA	GLRaV 1, 3, GVA	**Total**
	GLRaV 1	**4/22**		**2/4**	**2/20**	**12/26**		**7/22**	**27/94**
	GLRaV 2			0/4					0/4
100 L1	GLRaV 3		0/10	0/3	0/20		0/6	0/22	0/61
	GVA			0/2		**9/26**	0/6	**6/22**	**15/56**
	Test plants	22	10	4	20	26	6	22	110
	GLRaV 1	**29/68**		**4/5**	**12/56**	**25/55**		**26/47**	**96/231**
	GLRaV 2			0/5					0/5
50–100 L2	GLRaV 3		0/42	0/3	0/56		0/15	0/47	0/163
	GVA			**2/3**		**21/55**	**1/15**	**19/47**	**43/120**
	Test plants	68	42	5	56	55	15	47	288
7–50 L2 *	GLRaV 1	0/10							0/10
50–100 L1–L2	Healthy sources								0/49
3–70 L2 *	Healthy sources								0/4

**Table 5:** Virus transmission rates by vineyard-collected L1 and L2 of *Parthenolecanium corni* according to locality and to virus associations in plant sources, after transmission experiments. Number of positive vines/number of inoculated vines. Transmission events are highlighted in bold.

		**Viruses in Source plants**					
Locality	Cultivar	GLRaV 1	GLRaV 3	GLRaV 1, GVA	GLRaV 1, 3	GLRaV 3, GVA	GLRaV 1, 3, GVA
Bennwihr	Pinot noir		0/14		0/1		**1/1**
Ihringen	Kerner		0/12				
Kientzheim	Riesling			**7/10**			
Turckheim	Sylvaner	**1/2**		**6/9**			
Nothalten	Riesling	**33/88**	0/17	**27/65**	**16/78**	**1/21**	**32/68**
Ribeauvillé	Riesling		0/1	**1/2**			
Total transmission rates		**34/90**	0/44	**41/86**	**16/79**	**1/21**	**33/69**
GLRaV 1 % transmission		38%	-	48%	20%	-	48%
Virus % transmission		38%	0%	48%	20%	5%	48%

**Table 6:** Transmission rates of GLRaV 1 and GVA by L1 and L2 of *Parthenolecanium corni*, according to cultivars of recipient vines, after transmission experiments. In brackets, number of positive recipient vines/number of inoculated vines.

Recipient	**Transmission rates**	
cultivar	GLRaV 1	GVA
Pinot noir P 114	35% (18/52)	21% (11/52)
Pinot noir P 115	28% (64/229)	12% (27/229)
Pinot noir	44% (31/71)	24% (17/71)
Pinot blanc	32% (9/28)	14% (4/28)
Muscat Ottonel	5% (1/19)	0% (0/19)

## Data Availability

Data supporting the results reported are available on request.

## Supplementary Materials

The following supporting information can be downloaded at: https://www.mdpi.com/article/10.3390/v14122679/s1, Table S1: Comparison between virus contents in leaves from canes of the same source grapevine and their respective *Parthenolecanium corni* L1 nymphs (50 LI per batch) before a set of transmission experiments, and in leaves from recipient grapevines one year after inoculation.
