# Supplementary material for: Transmission of Grapevine Leafroll-Associated Viruses and Grapevine Virus A by Vineyard-Sampled Soft Scales (Parthenolecanium corni, Hemiptera: Coccidae)
Source: Viruses. 2022 Nov 29;14(12):2679. doi: 10.3390/v14122679 (PMC9784781; doi:10.3390/v14122679)
Supplement: Supplementary file 1 [file viruses-14-02679-s001.zip › viruses-1956038-supplementary.pdf]

viruses MDPI

Table S1. Comparison between virus contents in leaves from canes of the same source grapevine and their respective *Parthenolecanium corni* L1 nymphs (50 LI per batch) before a set of transmission experiments, and in leaves from recipient grapevines one year after inoculation.

					Viruses detected in	
Source vine			Viruses detected in source vines		L1 nymphs	Recipient vines
row	stock	twig	using ELISA	using RT-PCR	using RT-PCR	using ELISA
4	7	1		GLRaV 1, GVA	GLRaV 1, GVA	GLRaV 1, GVA
4	7	2	GLRaV 1	GLRaV 1, GVA	GLRaV 1, GVA	GLRaV 1, GVA
4	7	3	and GVA	GLRaV 1, GVA	GLRaV 1, GVA	dead
7	2	1		GLRaV 1, GVA	GLRaV 1, GVA	GLRaV 1, GVA
7	2	2	GLRaV 1	GLRaV 1, GVA	GLRaV 1, GVA	GLRaV 1, GVA
7	2	3	and GVA	GLRaV 1, GVA	GLRaV 1, GVA	dead
7	2	4		GLRaV 1, GVA	GLRaV 1, GVA	GLRaV 1, GVA
5	4	1,1		GLRaV 1, GVA	GLRaV 1, GVA	negative
5	4	1,2	GLRaV 1	GLRaV 1, GVA	GLRaV 1, GVA	GLRaV 1, GVA
5	4	2	and GVA	GLRaV 1, GVA	GLRaV 1, GVA	negative
5	4	3		GLRaV 1, GVA	GLRaV 1, GVA	negative
6	2	1		GLRaV 1, GVA	GLRaV 1, GVA	negative
6	2	2	GLRaV 1	GLRaV 1, GVA	GLRaV 1	GLRaV 1, GVA
6	2	3	and GVA	GLRaV 1, GVA	GLRaV 1	negative
5	58	1	GLRaV 1	GLRaV 1, 3	GLRaV 1, 3	negative
5	58	2	and 3	GLRaV 1, 3	GLRaV 1, 3	GLRaV 1
5	72	1	GLRaV 1	GLRaV 1	negative	negative
5	72	2	and 3	GLRaV 1	negative	GLRaV 1
6	35	1		GLRaV 1	GLRaV 1	negative
6	35	2	GLRaV 1	GLRaV 1	GLRaV 1	negative
6	35	3		GLRaV 1	GLRaV 1	GLRaV 1
6	36	1	GLRaV 1	GLRaV 1	GLRaV 1	negative
6	36	2		GLRaV 1	GLRaV 1	negative
8	4	1	negative	GLRaV 1	GLRaV 1	GLRaV 1
8	4	2		GLRaV 1	GLRaV 1	dead
6	50	1	negative	negative	negative	negative
6	50	2	control	negative	negative	negative
